# Supplementary material for: Identification of novel protective loci for executive function using the trail making test part B in the Long Life Family Study
Source: bioRxiv. 2025 Jul 11:2025.07.08.663708. Preprint. [Version 1] doi: 10.1101/2025.07.08.663708 (PMC12265606; doi:10.1101/2025.07.08.663708)
Supplement: Supplement 1 [file media-1.docx]

**Identification of novel protective loci for executive function using the trail making test part B in the Long Life Family Study**

Lihua Wang^1^, Katherine Tanner^1^, Stacy L. Andersen^2^, Stephanie Cosentino^3,4^, Vaha Akbary Moghaddam^1^, E. Warwick Daw^1^, Jason A. Anema^1^, Shiow Jiuan Lin^1^, Acharya Sandeep^5^, Michael Province^1^, Mary K. Wojczynski^1^

^1^Division of Statistical Genomics, Washington University School of Medicine, St. Louis, MO, USA

^2^Section of Geriatrics, Department of Medicine, Boston University Chobanian & Avedisian School of Medicine, Boston, Massachusetts, USA

^3^Department of Neurology, Columbia University Irving Medical Center, New York City, New York, USA

^4^The Taub Institute for Research on Alzheimer’s disease and the Aging Brain, Columbia University Irving Medical Center, New York City, New York, USA

^5^Division of Computational & Data Sciences, Washington University School of Medicine, St. Louis, MO, USA

**Figure S1. GWLS of TMT-B adjusted by TMT-A**

Plots of linkage analyses across 22 chromosomes. The x-axis denotes the physical distance in base pairs by 22 chromosomes. The y-axis indicates the LOD Score of the linkage analyses. LOD score is 4.4451 at chromosome 7, 1.3552 at chromosome 11, and 2.7687 at chromosome 15.


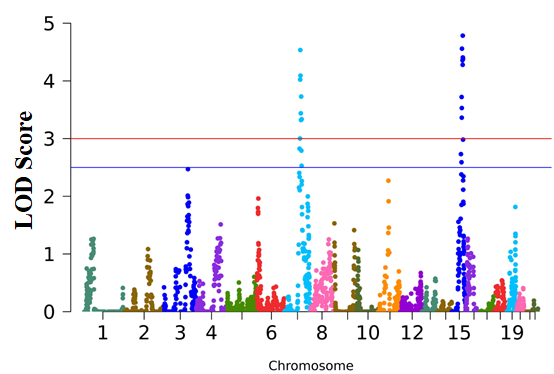


**Figure S2. GWLS of TMT-B adjusted by cognitive status**

Plots of linkage analyses across 22 chromosomes for TMT-B adjusted by dosage of cognitive status (Alzheimer’s Disease coded 1 for yes and 0 for no, and mild cognitive impairment coded 1 for yes and 0 for no). The x-axis denotes the physical distance in base pairs by 22 chromosomes. The y-axis indicates the LOD Score of the linkage analyses. LOD score is 4.5353 at chromosome 7, 2.2695 at chromosome 11, and 4.7841 at chromosome 15.


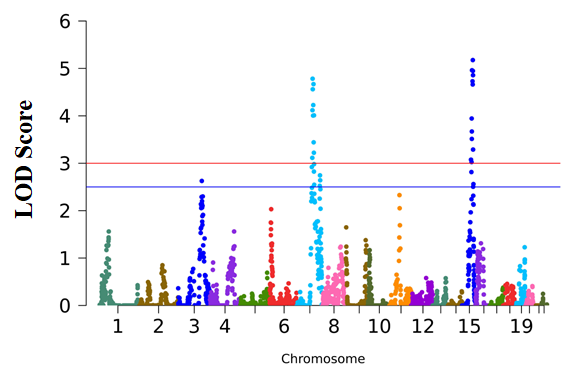


**Figure S3. GWLS of TMT-B adjusted by APOE**

Plots of linkage analyses across 22 chromosomes for TMT-B adjusted by dosage of APOE Ɛ2 (Ɛ2/Ɛ2 coded as 2, one copy of Ɛ2 coded as 1, and zero copy of Ɛ2 coded as 0) and dosage of APOE Ɛ4 (Ɛ4/Ɛ4 coded as 2, one copy of Ɛ4 coded as 1, and zero copy of Ɛ4 coded as 0). The x-axis denotes the physical distance in base pairs by 22 chromosomes. The y-axis indicates the LOD Score of the linkage analyses. LOD score is 4.7832 at chromosome 7, 2.3280 at chromosome 11, and 5.1762 at chromosome 15.


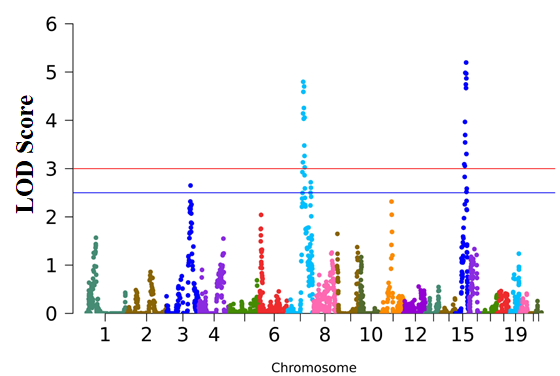


**Figure S4. GWLS of TMT-B adjusted by APOE**

Plots of linkage analyses across 22 chromosomes for TMT-B adjusted by dosage of APOE Ɛ2 (Ɛ2/Ɛ2 coded as 2, one copy of Ɛ2 coded as 1, and zero copy of Ɛ2 coded as 0). The x-axis denotes the physical distance in base pairs by 22 chromosomes. The y-axis indicates the LOD Score of the linkage analyses. LOD score is 4.7984 at chromosome 7, 2.3164 at chromosome 11, and 5.1971 at chromosome 15.


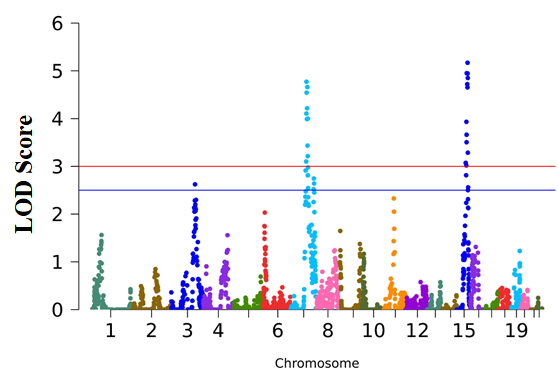


**Figure S5. GWLS of TMT-B adjusted by APOE**

Plots of linkage analyses across 22 chromosomes for TMT-B adjusted by dosage of APOE Ɛ4 (Ɛ4/Ɛ4 coded as 2, one copy of Ɛ4 coded as 1, and zero copy of Ɛ4 coded as 0). The x-axis denotes the physical distance in base pairs by 22 chromosomes. The y-axis indicates the LOD Score of the linkage analyses. LOD score is 4.7712 at chromosome 7, 2.3280 at chromosome 11, and 5.1697 at chromosome 15.
